# High-resolution data-driven model of the mouse connectome

**DOI:** 10.1162/netn_a_00066

**Published:** 2018-12-01

**Authors:** Joseph E. Knox, Kameron Decker Harris, Nile Graddis, Jennifer D. Whitesell, Hongkui Zeng, Julie A. Harris, Eric Shea-Brown, Stefan Mihalas

**Affiliations:** Allen Institute for Brain Science, Seattle, Washington, USA; Applied Mathematics, University of Washington, Seattle, Washington, USA; Applied Mathematics, University of Washington, Seattle, Washington, USA; Computer Science and Engineering, University of Washington, Seattle, Washington, USA; Allen Institute for Brain Science, Seattle, Washington, USA; Allen Institute for Brain Science, Seattle, Washington, USA; Allen Institute for Brain Science, Seattle, Washington, USA; Allen Institute for Brain Science, Seattle, Washington, USA; Allen Institute for Brain Science, Seattle, Washington, USA; Applied Mathematics, University of Washington, Seattle, Washington, USA; Allen Institute for Brain Science, Seattle, Washington, USA; Applied Mathematics, University of Washington, Seattle, Washington, USA

**Keywords:** Connectome, Whole-brain, Mouse

## Abstract

Knowledge of mesoscopic brain connectivity is important for understanding inter- and intraregion information processing. Models of structural connectivity are typically constructed and analyzed with the assumption that regions are homogeneous. We instead use the Allen Mouse Brain Connectivity Atlas to construct a model of whole-brain connectivity at the scale of 100 μm voxels. The data consist of 428 anterograde tracing experiments in wild type C57BL/6J mice, mapping fluorescently labeled neuronal projections brain-wide. Inferring spatial connectivity with this dataset is underdetermined, since the approximately 2 × 10^5^ source voxels outnumber the number of experiments. To address this issue, we assume that connection patterns and strengths vary smoothly across major brain divisions. We model the connectivity at each voxel as a radial basis kernel-weighted average of the projection patterns of nearby injections. The voxel model outperforms a previous regional model in predicting held-out experiments and compared with a human-curated dataset. This voxel-scale model of the mouse connectome permits researchers to extend their previous analyses of structural connectivity to much higher levels of resolution, and it allows for comparison with functional imaging and other datasets.

## Author Summary

Anatomical tracing experiments can provide a wealth of information regarding connectivities originating from the injection sites. However, it is difficult to integrate all this information into a comprehensive connectivity model. In this study we construct a high-resolution model of the mouse brain connectome using the assumption that connectivity patterns vary smoothly within brain regions, and we present several extensions of this model. We believe that this higher resolution connectome will be of great use to the community, enabling comparisons with other data modalities, such as functional imaging and gene expression, as well as for theoretical studies.

## INTRODUCTION

Brain network structure, across many spatial scales, plays an important role in facilitating and constraining neural computations. Models of structural connectivity have been used to investigate the relationship with functional connectivity, to compare brain structures across species, and more (Laramée & Boire, 2015; Sethi, Zerbi, Wenderoth, Fornito, & Fulcher, 2017; Stafford et al., 2014; X.-J. Wang & Kennedy, 2016). However, most of our knowledge of neuronal network connectivity is limited to either a detailed description of small systems (Bock et al., 2011; Glickfeld, Andermann, Bonin, & Reid, 2013; Kleinfeld et al., 2011; White, Southgate, Thomson, & Brenner, 1986) or a coarse description of connectivity between larger regions (Felleman & Van Essen, 1991; Sporns, 2010). In between these two extremes is *mesoscopic* structural connectivity: a coarser scale than that of single neurons or cortical columns but finer than whole-brain regions (Bohland et al., 2009). Facilitated by new tracing techniques, image-processing algorithms, and high-throughput methods, mesoscale data with partial to full brain coverage exist in animals such as the fly (Jenett et al., 2012; Shih et al., 2015) and mouse (Gămănuţ et al., 2018; Oh et al., 2014; Zingg et al., 2014), and such data are being collected from other model organisms such as rat (Bota, Dong, & Swanson, 2003) and marmoset (Majka et al., 2016).

We present a scalable regression technique for constructing spatially explicit mesoscale connectivity from anterograde tracing experiments. Specifically, our model estimates the projection strength between every pair of approximately 2 × 10^5^ cubic voxels, each 100 μm wide, in the Allen Mouse Brain Common Coordinate Framework (CCF v3). The CCF is a fully annotated reference atlas space with structure/region delineations. We use data from the Allen Mouse Brain Connectivity Atlas (Oh et al., 2014), a large dataset of viral tract-tracing experiments performed across many regions of the mouse brain. All of the data processing scripts are publicly available at https://github.com/AllenInstitute/mouse_connectivity_models (Knox, 2018).

In these mesoscale anterograde tracing experiments, a tracer virus (recombinant adeno-associated virus) is first injected into the brain. The virus infects neurons at the site of injection and causes them to express enhanced green fluorescent protein (eGFP) in their cytoplasm, including throughout the entire length of their axons. Brains and labeled axons are imaged with blockface serial two-photon tomography (Ragan et al., 2012) throughout the entire rostral-to-caudal extent of the brain, resulting in an aligned stack of 2-D images that can easily be transformed to 3-D space. Each brain contains one source injection only. Every image series is registered to the 3-D CCF, using a combination of global affine and local transformations (Kuan et al., 2015).

Combining many experiments with different injection sources in the same 3-D space reveals the set of pathways that connect those sources throughout the brain, the ingredients of a “connectome.” This requires combining data across multiple animals, which appears justified at the mesoscale (Bohland et al., 2009; Oh et al., 2014). Previous mouse connectome models were constructed with the assumption that regions are homogeneous (Gămănuţ et al., 2018; Oh et al., 2014; Ypma & Bullmore, 2016). While these have proven useful, they depend on predefined regional parcellations and describe connectivity at a region-limited level of resolution. Here, we go beyond the regional approach and construct a model of the whole-brain connectivity at the scale of 100 μm voxels. Previously, K. D. Harris, Mihalas, and Shea-Brown (2016) formulated a regularized, structured regression problem for inferring voxel connectivity. This model was applied to Allen Mouse Brain Connectivity Atlas data in the visual cortex, outperforming a regional model in prediction of held-out experiments.

Here, we extend the voxel approach from the visual cortex to the full mouse brain, while also simplifying the mathematical model for computational efficiency. Our model relaxes the assumption of homogeneity of connections within a region and instead assumes smoothness across major brain divisions. We model the connectivity at each source voxel as the weighted average of the projection patterns of nearby injections, where the weights are a monotonically decreasing nonlinear function of distance to the injection centroid. We fit the parameters of the model using nested cross-validation with held-out injection experiments. The new voxel-scale model generally outpredicts a homogeneous regional model, as measured both by cross-validation error and when compared with a human-curated dataset.

## RESULTS

### Spatial Method to Infer a Voxel Connectome

We consider the problem of fitting a weighted, directed, adjacency matrix that contains the connection strength between any pair of points in the brain. We use *n* cubic voxels, 100 μm across, to discretize the brain volume. Our goal is then to find a matrix *W* ∈ ℝ_≥0_^*n*×*n*^ that accurately captures voxel-voxel connection strength. We assume there exists some underlying matrix *W* that is common across animals. Each experiment can be thought of as an injection *X*, and its projections *Y*, where *X*, *Y* ∈ ℝ^*n*^, and we want to find *W* so that *Y* ≈ *WX*, that is, we want to solve a multivariate regression problem. The details of all procedures are found in Methods.

**Figure 1.**
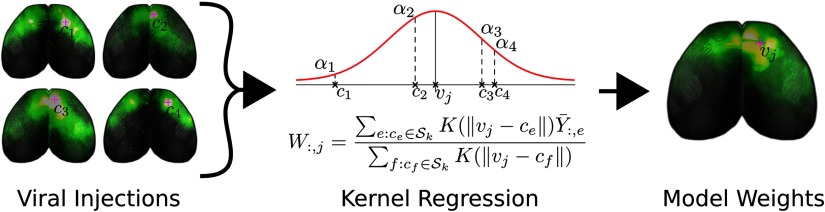
Cartoon illustrating our method of connectome inference. We combine the information from viral tracing experiments with different injection sites into a model of voxel structural connectivity. To predict the weight of projections *W*_*ij*_ from the *j*th voxel *v*_*j*_ to the *i*th voxel, we take an average of nearby injections where the *e*th experiment’s projection ${\overset{-}{Y}}_{ie}$ is weighted by a factor proportional to *K*(∥*v*_*j*_ − *c*_*e*_∥), *c*_*e*_ is that injection’s center of mass, and *K*(⋅) is the kernel.

We adopt a spatial weighting technique to combine information from multiple experiments into one matrix, the outline of which is shown in Figure 1. As in K. D. Harris et al. (2016), we assume that the connectivity from any given source voxel varies smoothly as a function of distance: Columns *W*_:,*i*_ and *W*_:,*j*_ should be similar if the distance between voxels *i* and *j* is small. We make the mathematically simplifying assumption that the projections we observe from a given experiment come from the center of mass of the injection *c*_*e*_. This allows us to employ kernel regression to approximate the connectivity from a given voxel *v* as the distance-weighted sum of injections in the major brain division containing *v*. We also expect the connectivity could change sharply between the boundaries of high-level brain structures. For example, we know that projections arising from the thalamus and hypothalamus can be very different, even though some areas within these divisions are near each other at the borders. To account for this, we chose a partition of the brain into 12 nonoverlapping *major brain divisions* or *coarse structures* defined in the CCF. The major brain divisions are isocortex, olfactory areas, hippocampal formation, cortical subplate, striatum, pallidum, thalamus, hypothalamus, midbrain, pons, medulla, and cerebellum.

### Voxel-Scale Model Compared With a Regionally Homogeneous Model

Previously, Oh et al. (2014) obtained a regional mouse connectome by integrating the injection and projection data over regions and fitting a region-by-region matrix with nonnegative least squares. Here, we recomputed this matrix (see Methods) and compared it with a regionalized version of the voxel connectivity. To avoid confusion between the two models, we call the regional connectome the *homogeneous* model, because it assumes homogeneity across anatomical structures. We call our new model, when it has been averaged over regions, the *regionalized* voxel model. In short, we chose 291 gray matter regions, which are intermediate level in the CCF, and recomputed the homogeneous model. The abbreviations of all CCF regions mentioned in this paper are given in Table S1 (Knox et al., 2019). To generate the regionalized voxel model, voxel connectivity was integrated and averaged over regions to produce regionalized weights (see Methods for details).

**Figure 2.**
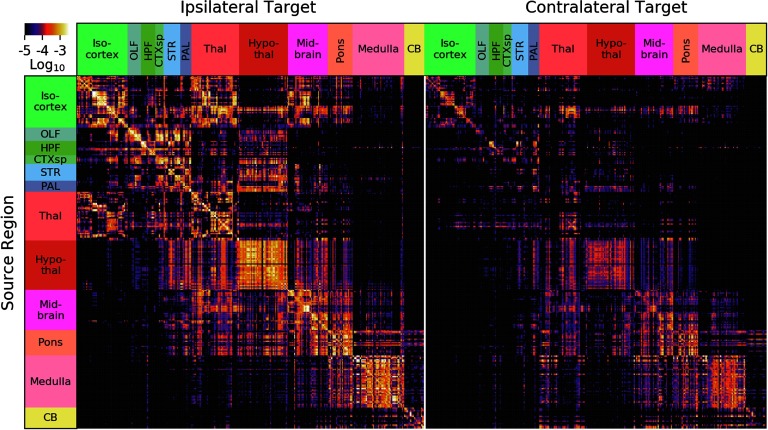
Whole-brain normalized connection density obtained from the regionalized voxel model. We show 291 gray matter regions divided into 12 major brain divisions. For visualization purposes, sources are shown on the rows and targets on the columns, the opposite convention as the mathematics in the text (*W*^⊺^ is pictured). The similarity between rows, for example in hypothalamus, is driven both by biological similarity and as a result of the model’s interpolation in the sources. The similarity between columns is the result of correlations in the data, as the model does not interpolate in target space.

**Figure 3.**
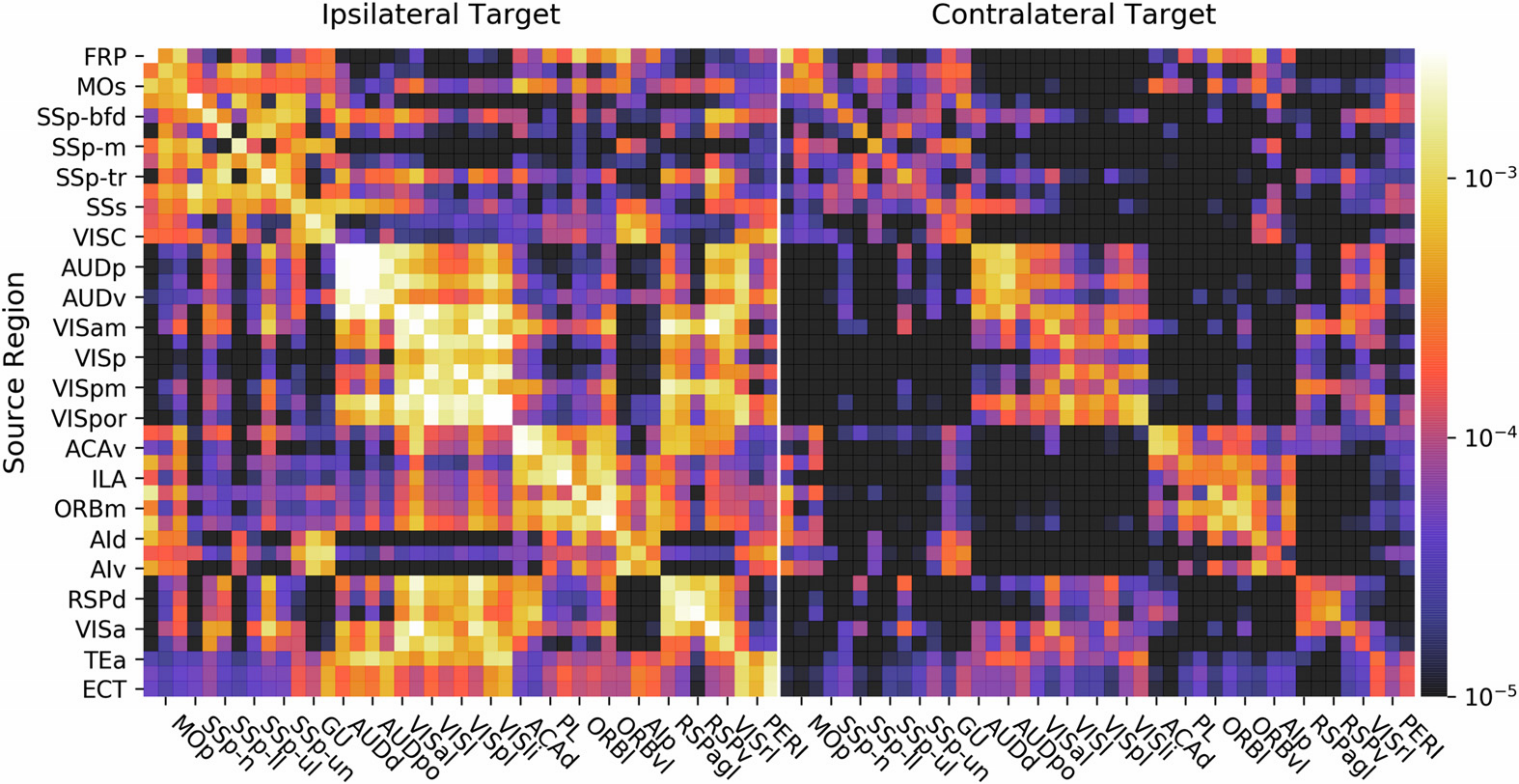
Isocortex normalized connection density from the regionalized voxel model. Again, we show sources as rows and targets as columns (*W*^⊺^ is pictured) for visualization.

In Figure 2, there is a depiction of the whole-brain regionalized weights and, in Figure 3, the regionalized weights for isocortex. Note that, for visualization purposes, we depict sources as rows and targets as columns (*W*^⊺^), the opposite of our mathematical convention.

A number of features are evident in Figures 2 and 3. First, there are patterns that arise from our smoothness assumption. The predicted projection patterns from a certain source voxel are distance-weighted averages of injections nearby that voxel (see Methods and Figure 1). Therefore, the method we employ smooths in the source space only. The vertical banded structures (for example, the column near the right side of the medulla division in Figure 2) are due to smoothing in source but not target regions, which makes the rows of *W*^⊺^ corresponding to nearby source voxels similar. In Figure 3, note that the rows PERI, ECT, and TEa (the bottom three rows) and AUDv (upper middle) match closely. All four of these regions are very close to each other on the posterior, ventrolateral part of the cortex, so that smoothing causes them to be correlated. Also notice that, while the rows corresponding to these regions as sources are very similar, the columns corresponding are not nearly as much so. This highlights that our model interpolates only in the sources and not the targets.

The second feature that is evident is the presence of blocks of strongly interconnected regions. These correspond to modules in the network, or regions that are more highly connected to each other than they are to the rest of the network. This is explored further in J. A. Harris et al. (2018).

In Table 1, we show the results of comparing homogeneous and voxel models. We fit the models using nested leave-one-out cross-validation. This allows us to evaluate both the voxel-scale and regionalized models’ error when predicting held-out data. We report mean squared error relative to the average squared norm of the prediction and data, which can be between 0 and 200%. See Equation 4 and the description in the Methods. The model validation and training errors (goodness of fit, shown in parentheses) are reported at both voxel (Voxel MSE_rel_) and regional (Region MSE_rel_) levels. Additionally, we compare model performance using a subset of experiments in which a given source region received at least three replicate injections. Without another injection, the only information about that region’s projections would come from our smoothness assumption interpolating nearby regions’ patterns. The computed relative error MSE_rel_ for this dataset of replicated injections we call the “power to predict” (Region PTP), and it tends to be lower than Region MSE_rel_ across divisions.

**Table 1.** Table of cross-validated model errors, comparing the voxel model and the regionally homogeneous model. In each case the training error is in parentheses. Voxel MSE_rel_ refers to relative error, Equation 4, at the voxel level. This measure approximates the data normalized MSE for small errors, but is bounded to maximum of MSE_rel_ = 200%, which is achieved if either *Y*^true^ or *Y*^pred^ is 0 and the other is not (see Methods). Region MSE_rel_ is the error found after regionalizing the voxel model prediction. PTP (“power to predict”) computes MSE_rel_ for only those held-out experiments where there was another injection in that region which was not used for fitting.

**Major division**	**Model**	**Voxel MSE_rel_**		**Region MSE_rel_**		**Region PTP**	
**Isocortex**	Voxel	66%	(22%)	34%	(11%)	32%	(9%)
	Homogeneous	–	–	37%	(20%)	33%	(17%)
**Olfactory areas**	Voxel	82%	(17%)	41%	(6%)	40%	(6%)
	Homogeneous	–	–	50%	(9%)	44%	(9%)
**Hippocampus**	Voxel	92%	(20%)	56%	(17%)	52%	(17%)
	Homogeneous	–	–	46%	(51%)	48%	(51%)
**Cortical subplate**	Voxel	114%	(40%)	95%	(47%)	93%	(25%)
	Homogeneous	–	–	111%	(2%)	99%	(2%)
**Striatum**	Voxel	103%	(8%)	45%	(2%)	40%	(1%)
	Homogeneous	–	–	53%	(23%)	53%	(24%)
**Pallidum**	Voxel	100%	(5%)	64%	(3%)	45%	(7%)
	Homogeneous	–	–	85%	(3%)	74%	(2%)
**Thalamus**	Voxel	115%	(14%)	77%	(10%)	70%	(12%)
	Homogeneous	–	–	92%	(12%)	86%	(12%)
**Hypothalamus**	Voxel	69%	(46%)	48%	(32%)	37%	(9%)
	Homogeneous	–	–	62%	(5%)	77%	(7%)
**Midbrain**	Voxel	89%	(32%)	42%	(14%)	37%	(13%)
	Homogeneous	–	–	44%	(14%)	39%	(14%)
**Pons**	Voxel	98%	(9%)	66%	(6%)	61%	(6%)
	Homogeneous	–	–	89%	(27%)	87%	(27%)
**Medulla**	Voxel	96%	(33%)	51%	(18%)	49%	(21%)
	Homogeneous	–	–	58%	(3%)	54%	(2%)
**Cerebellum**	Voxel	177%	(9%)	78%	(2%)	75%	(7%)
	Homogeneous	–	–	90%	(4%)	63%	(5%)

From Table 1, we see that the relative training and validation errors are higher when evaluating error in the voxel space. This makes sense, because this error captures mistakes we make in predicting spatial patterns of projections at the voxel, subregional level. That task is much more difficult than predicting regional patterns. The lowest voxel errors are in isocortex, hypothalamus, and olfactory areas, whereas the highest are in cerebellum, thalamus, and cortical subplate. At the regional level we compare both the homogeneous model and the regionalized voxel model, which uses the voxel connectome to make a prediction that is then integrated across each region. At the regional level, our voxel model has lower regional validation errors than the homogeneous model in 11/12 major brain divisions. The training error is lower in 7/12 cases, since assuming smoothness is less biased than regional homogeneity. The regional PTP of the voxel model is lower than the PTP of the homogeneous model in 10/12 cases. Results for training PTP are better for the regionalized model in 6/12.

In general, we find the highest errors (for either model) in cortical subplate, which is the smallest major division and has the largest distance between injections. The voxel and regional test errors are correlated with the mean minimum distance between voxels and injection centers of mass within each major brain division (correlation coefficients of 0.50 and 0.68, respectively). We find the smallest errors are in isocortex, which has the largest number of replicated experiments. The summary statistics of our data by major brain division are summarized in Table S2 (Knox et al., 2019).

### Visualizing Voxel-Scale Connectivity: Cortico-Cortical Virtual Injections

Visualization of our model faces two challenges: The matrix *W* contains *n* × *n* = *O*(10^11^) entries, and represents dense connectivities between 3-D spatial structures. In order to address these challenges, we generated “virtual injections,” which are just the predicted projections from a given source voxel of interest. These virtual injections allow us to visualize the average projections from voxels of our choosing. This process is efficient, because the matrix *W* is formed from *m* rank one components, so we never have to form the entire matrix. Standard tools, such as volume rendering and projection, can then be applied to visualize the model’s predictions.

In order to visualize model predictions in the isocortex we make use of a curved cortical coordinate system. This coordinate system defines two dimensions over the surface of the cortex and one that is composed of steepest-descent paths from the pia surface to white matter. By projecting model predictions along these paths, we can generate 2-D cortical projection maps that are faithful to the boundaries of isocortical regions.

**Figure 4.**
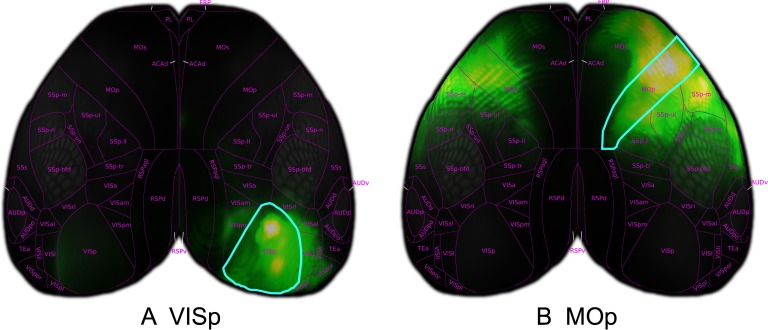
Model predicted cortico-cortical projections from virtual injections into the entire primary visual area (VISp) and primary motor area (MOp).

We display two such projections in Figure 4. Here, we visualize the average over the columns of the matrix *W* corresponding to the projections from two isocortical regions, marked by a cyan outline. We observe strong ipsilateral projections to nearby areas, as expected from the experimental data. For instance, primary visual area VISp has a number of local projections to higher visual areas, Figure 4A, and primary motor cortex MOp exhibits strong connectivity to secondary motor and somatosensory areas, Figure 4B. We also observe weaker contralateral projections across the midline in a similar pattern to the ipsilateral hemisphere.

### Weight Distribution and Its Distance Dependence

**Figure 5.**
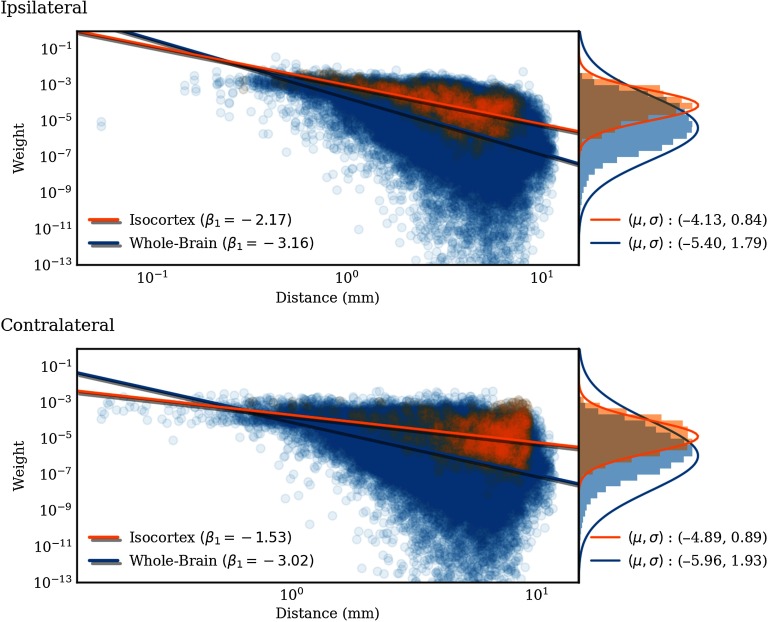
Normalized connection density produced by the regionalized voxel model (log scale) plotted against interregion distance for 291 regions in the whole-brain (blue) and for only cortico-cortical connections (orange). The lines are linear least squares of the form log_10_(weight) = *β*_1_ log_10_(distance) + *β*_0_. The histograms on the right side show the distributions of weights as well as Gaussian fits of the log_10_(weight) distribution. Note that the standard deviations are biased because of small weight outliers.

We compared multiple models for the distribution of connection weights: lognormal (as has been reported in Markov et al., 2014; Q. Wang, Sporns, & Burkhalter, 2012), inverse gamma, exponential, and normal. We separately construct these models for ipsilateral and contralateral connections for the entire brain and for connections within isocortex. For all these weight distributions, the best fit is for a lognormal distribution, as selected by Bayesian information criterion (BIC). The BIC is a pseudo-likelihood that penalizes the number of model parameters. See Table S3 (Knox et al., 2019). However, the results from the Kolmogorov-Smirnov test show that the fitted lognormal distributions fail to be statistically similar to the weight distributions for any division of the connections at *α* = 0.05 significance. Additionally, the logarithmically transformed weights fail to pass the Shapiro-Wilk test for normality at the same level of significance. This is because the log-transformed weights, depicted on the right-hand side of Figure 5, exhibit a skewed distribution.

We have previously seen that a heterogeneous set of connections can be better fit by a mixture of lognormal distributions (Oh et al., 2014). In a similar manner, we find the logarithmically transformed weights are best fit by a multiple component Gaussian mixture model (GMM). See Table S4 (Knox et al., 2019). The number of components was selected to minimize the BIC. This resulted in a five-component GMM for the whole brain, and a two- to three-component GMM for cortico-cortical connections. With the exception of two components in each of the whole-brain mixture models, the components have similar valued weights, suggesting that different regions contribute to a nonhomogeneous distribution of connection weights across the brain. However, it could also be the case that the empirical distribution of log-transformed weights is well modeled by a skewed unimodal distribution.

In Figure 5, we show the dependence of extant connection weights on distance. We compared an exponential (Ercsey-Ravasz et al., 2013) and a power law fit. Using the Levenberg- Marquardt algorithm to fit each nonlinear least squares problem, the root mean squared training error (RMSE) was found to be slightly smaller for the power-law fit, but similar for both.

### Model Performance Compared With Anatomical Data

**Figure 6.**
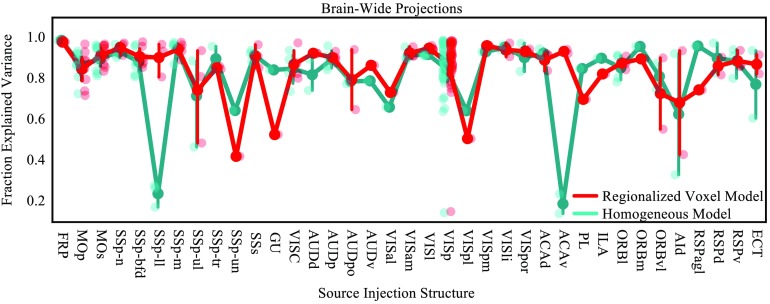
Fraction of whole-brain projection volume from cortical sources explained by model weights for the regionalized voxel model (red) and the homogeneous model (cyan). Each of the 37 cortical source regions is plotted on the x-axis, and the transparent points are for individual experiments. Darker points and lines indicate the mean and 95% confidence interval across all injections delivered to that source.

To evaluate how closely the model predictions aligned with experimental data, we compared the model weights from the homogeneous model and the regionalized voxel models with projection data for each of the 128 injections into the isocortex in wild type mice from the Allen Mouse Brain Connectivity Atlas. For each injection experiment, we calculated the fraction of explained variance for the two models by taking the square of the Pearson correlation between the experimental normalized projection volume and the model weights in each of the 291 regions. Figure 6 shows the fraction of explained variance for each experiment grouped by source structure. Although the mean predicted variance across all sources was similar between the two models, the regionalized voxel model performed better overall (mean ± *SD* regionalized: 0.87 ± 0.13, homogeneous: 0.84 ± 0.17, *p* < 0.0001, paired *t* test), but note that each model outperformed the other for some sources.

To further explore the relationship between the model prediction and the experimental data, we also compared the predicted weights from both models with experimental data from a subset of experiments in which injection sites were more than 95% contained within a single source region. There were 36 experiments that met this criterion. Of these, 35 were in the isocortex and 1 was in the hippocampus, and this set contained in the following 10 sources (see Table S1, Knox et al., 2019): AUDp (1), ENTl (1), MOp (3), MOs (1), RSPd (1), SSp-bfd (1), SSp-m (4), SSp-n (1), SSs (1), and VISp (22). Note that in VISp we include four injections using the pan-excitatory Cre line Emx1-IRES-Cre mice, which have very similar projection patterns as wild type mice (J. A. Harris et al., 2018). Twelve of these experiments were manually checked for segmentation errors in the regions where nonzero projections were automatically flagged. When these manually annotated data were available, we multiplied the normalized projection volume by 1 for true positives or 0 for true negatives. See Figure S1 (Knox et al., 2019) and the description in Methods. For each of these 10 sources, we plotted the normalized projection volume from the experimental data along with the predicted weights from both models for targets in the ipsilateral isocortex.

**Figure 7.**
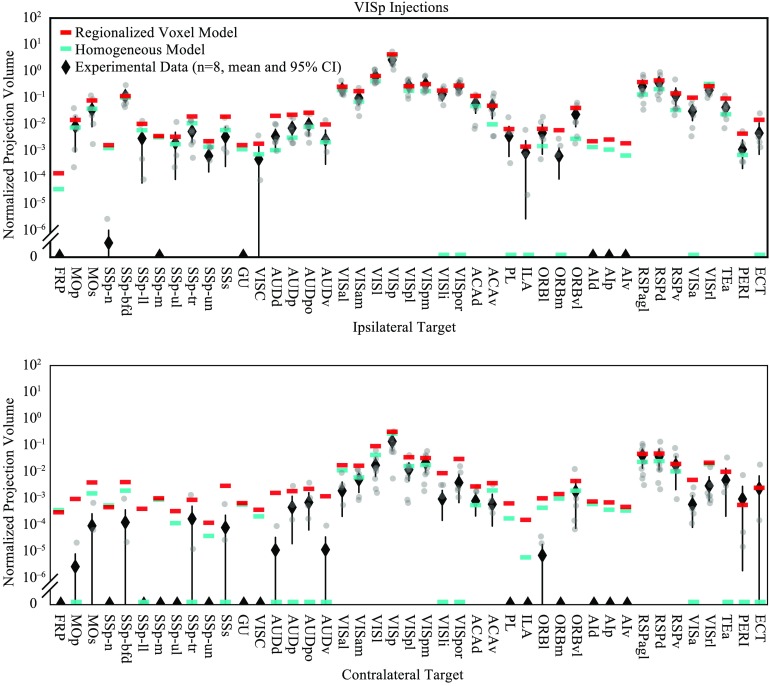
Normalized projection volume for VISp projections to ipsilateral (top) and contralateral (bottom) cortical targets in log scale. The raw data are compared with model predictions. Normalized projection volume from individual manually validated experiments (gray) and the mean and 95% confidence interval for each target are plotted as black diamonds. Note that many confidence intervals are smaller than the markers. Corresponding weights for VISp projections to each cortical target from the homogeneous model (cyan) and the regionalized voxel model (red) are overlaid.

Figure 7 shows the plot for VISp, where we had a total of 22 experiments of which eight were checked for true positives/true negatives at all cortical targets. Four injections into Emx1-IRES-Cre mice were included in the plot for VISp, although these were not used to fit the model. Only the eight experiments that were manually checked are included in the plot in the figure. The weights predicted by the regionalized voxel model were higher overall, but generally agreed with the experimental data as well as the homogeneous model predictions. The biggest difference between the two models was that the regionalized voxel model correctly predicted nonzero weights for several targets that were missed by the homogeneous model. All of the ipsilateral targets of VISp that had a weight of 0 in the homogeneous model were verified true positives in at least three of the eight experiments plotted in Figure 7. All of the contralateral targets except SSp-ll were verified true positives in at least two out of the eight experiments. The regionalized voxel model predicted nonzero weights for all of these true positive targets that were assigned a weight of 0 by the homogeneous model. Contralateral SSp-ll was a true negative in all eight experiments and was incorrectly assigned a weight by the regionalized voxel model but not the homogeneous model. Both models predicted small but nonzero weights for several other targets that were true negatives (ipsilateral: FRP, AId, AIp, and AIv; contralateral: FRP, SSp-n, SSp-m, SSp-ul, GU, VISC, PL, ILA, AId, AIp, AIv). Across all 10 sources that were checked, the regionalized voxel model tended to predict nonzero weights for connections that were assigned 0 weight in the homogeneous model.

Overall, we found the predictions of the regionalized voxel model to be more consistent with the data than the homogeneous model. Even when the predicted weight from the regionalized voxel model was incorrect, it was not off by a large margin. The homogeneous model often performs well, but when its predictions differ from the experimental data they are sometimes off by a very large margin. For example, the homogeneous model predicts zero weight for projections from ACAv to the caudoputamen (CP), but the experimental data show that CP is in fact the strongest projection target of ACAv with a normalized projection volume of 4.4 and 5.0 (unitless) in the two available experiments (compared with 0.05 ± 0.28, mean ± *SD* for all targets of ACAv across the two experiments). The homogeneous model makes the same error for SSp-ll, again assigning zero weight to projections in CP when the experimental data show that CP is the highest weight target of SSp-ll (normalized projection volume in CP = 3.2 ± 1.1, mean normalized projection volume in all targets = 0.03 ± 0.2, *n* = 3 experiments). This is reflected in the standard deviation for the fraction explained variance (0.17 for the homogeneous model vs. 0.14 for the regionalized voxel model), and is well illustrated in Figure 6, where the homogeneous model performs well overall, but very poorly for projections from SSp-ll and ACAv.

## DISCUSSION

In this study, we infer whole-brain connectivity at 100 μm voxel resolution from a set of brain-wide anterograde viral tracing experiments in young adult wild type C57BL/6J mice (Oh et al., 2014, http://connectivity.brain-map.org). The central assumption of extant voxel methods is that brain-wide projections from nearby neurons within a brain region vary smoothly. Such a method, and its application to the visual system, has been described by K. D. Harris et al. (2016). Eventually, we hope to improve those mathematical methods to solve the original source- and target-smoothed nonnegative regression problem at whole-brain scale. However, the current implementation of that problem is computationally costly and does not scale to the whole brain. These considerations led us to develop the simpler method presented here.

Our approach makes two simplifying assumptions (see Methods): First, we assume the injection is delivered into just the center of mass voxel, rather than the entire injection volume. This is the opposite extreme of the regionally homogeneous assumption. Second, we perform interpolation in only the source space, rather than also in the target space. With these changes, the model of K. D. Harris et al. (2016) becomes, essentially, the method we present here, because of the well-known “kernel trick” (Wahba, 1990). This connection is inexact because of boundary effects, the particular choice of kernel, and the use of Nadaraya-Watson rather than regression coefficients.

The tracing data that forms the basis of this study is based on anterograde viral tracers (Oh et al., 2014). The viral tracing methods used to generate the Allen Mouse Brain Connectivity Atlas dataset result in two limitations affecting our ability to resolve connections. One limitation comes from the size of the injections, which have a typical radius of 0.3 mm. Table S2 (Knox et al., 2019) provides the volume distribution through different brain regions. An even stronger limit comes from the distances between a voxel and the center of mass of an injection being typically 0.5 mm. Table S2 reports this average in the “injection distance” column. This distance is the consequence of the number of injections (491, of which we select 428 as described in Methods) being much smaller than the number of source voxels (2.5 × 10^5^). The connections originating from a voxel have to be inferred from sources on average 0.5 mm away, which average information over a radius of 0.3 mm. Spatial averaging is thus necessary for these data.

Voxel models were fit and analyzed separately for a set of 12 major brain divisions. These structures represent a coarse level of anatomical parcelation of mammalian brains and have qualitatively different connection patterns. One could also take an agnostic approach and hope that the data reveal where these differences arise. Perhaps using a total variation or similar regularization that allows for piecewise constant patterns, or working with a factored *W* = *LR* and clustering the factors into different regions, could provide a tractable approach to this. Nonnegative matrix factorizations can also be interpreted as another way to find network modules, as was proposed in K. D. Harris et al. (2016). However, this both increases the mathematical difficulty and could require more experiments than are currently available.

We compared the voxel and homogeneous models’ ability to predict held-out injection experiments. Although the errors for both are relatively high (but see the Supporting Information (Knox et al., 2019) for a comparison with log-transformed errors), the voxel model on average performs better. However, if the homogeneous model is fit with 10 μm data, it can outperform the regionalized voxel model fit with 100 μm data (see the Supporting Information). We believe a good method to evaluate the model’s performance is to compare the predicted weights with a human-curated ground truth metric. We were able to make this comparison for a subset of injections well contained in 10 of 43 cortical sources. By comparing the models’ predictions with experiments, we found two main differences between the regionalized voxel model and the homogeneous model. First, and most importantly, the regionalized voxel model predicts very weak but nonzero connections that the homogeneous model assigns zero weight. This is due to the inherent tendencies of the models to increase sparsity (homogeneous model) or to decrease sparsity (regionalized voxel model). We verified some of the connections that were detected by the regionalized voxel model and not the homogeneous model as true positives, but others were true negatives that were incorrectly assigned a nonzero weight by the regionalized voxel model. Occasionally, the homogeneous model assigns a zero weight to very strong connections as with the projections to CP from ACAv and SSp-ll (Figure 6), but the regionalized voxel model is less susceptible to this type of error since it tends to decrease sparsity.

The other main difference between the two models was in the prediction of weights for small target structures. Because the regionalized voxel model is a linear smoother, it will tend to overpredict weak connections for targets near regions with high connectivity. Some examples of this behavior can be seen in Figure 7 where projection weight to AUDv, for example, is overestimated by the regionalized voxel model, likely because of its spatial position between AUDp and TEa which both receive strong projections from VISp. In choosing the appropriate model for an application, it is therefore important to consider that weak connections have higher uncertainty, and whether false positives or false negatives have a greater influence on the results.

We would like to emphasize that when analyzing this connectivity, especially from a graph theoretical perspective, one also has to be mindful of the correlations between connections originating from nearby sources that are introduced by the method. The spatial resolution of the connectivity is presented at 100 μm resolution. However, at source level, the average distance to the closest injection is typically 0.5 mm (see Table S2, Knox et al., 2019), which limits the resolution. Also, many graph statistics may not be well suited for studying such explicitly spatial graphs as ours.

Among multiple models for unimodal weight distributions, we found the lognormal being the best fit, in accordance with previous studies (e.g., Markov et al., 2014; Q. Wang et al., 2012), despite failing the Kolmogorov-Smirnov test. Following this analysis, we found that a mixture of normal distributions was an even better fit for the distribution of log weights (as in Oh et al., 2014). A possible explanation for this is that the mixture results from combining heterogeneous neuronal populations each with lognormal statistics, or that the log-transformed distribution is skewed.

We analyzed the distance dependence of the connection weights and found that a power law dependence is a marginally better fit than exponential (Ercsey-Ravasz et al., 2013), similar to the result of Rubinov, Ypma, Watson, and Bullmore (2015). It is interesting that the power is close to −2 for the cortex (ipsilateral), which can be roughly approximated as a 2-D “sheet,” and close to −3 for the entire brain, which is 3-D. However, the weak scaling we observe only holds over roughly 1.5 orders of magnitude, so we prefer not to speculate too much about this result.

The voxel model enables quantitative characterization of the structural connectivity of the mouse brain. It is a significant improvement over the previously published homogeneous linear model (Oh et al., 2014), with easily tractable mathematics compared with the earlier voxel proposal (K. D. Harris et al., 2016). It offers improved predictions at region level, but, more importantly, it provides the connectivity at a much higher spatial resolution. This new model provides the necessary basis for studies of large-scale network structure, enabling discovery of general organizational rules for brain-wide systems that consist of both local and long-distance connections. For example, J. A. Harris et al. (2018) performed an analysis of modularity in the wild type cortical subnetwork, employing the regionalized voxel model we present here. They found that the cortical network divides into 1–14 modules, depending on the clustering parameters, but found six stable modules that were characterized as prefrontal, anterolateral, somatomotor, visual, medial, and temporal. A better understanding of such structural rules will lead to more accurate predictions of the directions of information flow, constrained by anatomy, and can be used by researchers interested in questions of structure-function relationships in the mouse brain.

## METHODS

### Summary of Data

The data were taken from 491 experiments using wild type C57BL/6J mice. These data are available from the Allen Mouse Brain Connectivity Atlas at http://connectivity.brain-map.org/ (Oh et al., 2014). The brain is divided into a set of *s* = 12 *major brain divisions* at a high level of the CCF ontology. These major brain divisions are isocortex, olfactory areas, hippocampal formation, cortical subplate, striatum, pallidum, thalamus, hypothalamus, midbrain, pons, medulla, and cerebellum. We also consider a finer partition (lower in the ontology) into a set of *r* = 291 *regions*. The major brain divisions form a disjoint partition of the brain, as do the regions. These regions are each contained within a given major brain division.

We curated experiments to exclude those in which infected cell bodies are located in multiple major brain divsions. For example, we removed experiments with large injection volumes spanning multiple subcortical major brain divisions and subcortical injections with substantial leakage of the tracer in the overlying cortex. Additionally, we removed four experiments having very little to no long-distance projections (small projection volume outside of the injection location). Overall, of the 491 experiments, we removed 63 experiments resulting in a total of *m* = 428 included experiments. We summarize these experiments used to fit our connectome in Table S2 (Knox et al., 2019).

In our mathematical framework, the brain is a subset of ℝ^3^ that is discretized into a collection of *n* cubic voxels. Subsets of these voxels then correspond to the major brain division {𝒮_*i*_}_*i*=1_^*s*^ and regions {𝓡_*i*_}_*i*=1_^*r*^. Each voxel *i* maps to a location in the brain, which we denote by *v*_*i*_ ∈ ℝ^3^. Each injection tracing experiment produces an image stack (i.e., a 3-D image) of fluorescently labeled neurons and axons throughout the brain. The fluorescence signal is reported as injection density (fraction of fluorescing pixels per voxel for voxels in the annotated injection site) and projection density (fraction of fluorescing pixels per voxel outside of the injection site). For the *e*th experiment, let *X*_:,*e*_ and *Y*_:,*e*_ denote the length *n* vectors of injection density and projection density, respectively. We also compute voxel coordinates of the center of mass of the injection density *c*_*e*_ ∈ ℝ^3^. For our estimator, we also compute the normalized projection density, normalizing by the sum of the injection density, and denote this ${\overset{-}{Y}}_{:,e}$. Note that ${\overset{-}{Y}}_{:,e}=(Y_{:,e}+X_{:,e})/\sum_{v}X_{ve}$, since we also include the injection pattern in the normalized projection density. Thus, the experimental data are this collection ${\left\{(X_{:,e},Y_{:,e},{\overset{-}{Y}}_{:,e},c_{e})\right\}}_{e=1}^{m}$, of length *n* vectors as well as the injection centers of mass for each experiment.

### Multivariate Nonparametric Regression to Infer Voxel Connectivity

We consider the problem of fitting a nonnegative, weighted adjacency matrix *W* ∈ ℝ_≥0_^*n*×*n*^ that is common across animals. Entry *W*_*ij*_ is the estimated projection density of neurons in voxel *j* to voxel *i*, if one unit of injection density were delivered to voxel *j*. Each experiment consists of an injection *X*, and its projections *Y*, and we would like to find *W* so that so that *Y* ≈ *WX*. Uncovering the unknown *W* from multiple experiments (*X*_:,*e*_, *Y*_:,*e*_) for *e* = 1, …, *m* is then a multivariate regression problem. The unknown matrix *W* is a linear operator that takes images of the brain (injections) and returns images of the brain (projections).

Unlike the earlier work by K. D. Harris et al. (2016), we make two crucial simplifying approximations: First, we assume that in experiment *e* the injection is delivered to precisely one voxel, the injection center of mass *c*_*e*_. This removes the more difficult credit assignment problem of which voxels within each injection site contribute which projections. The method of K. D. Harris et al. (2016) solved this problem by linear regression, essentially “dividing out” the injection correlations across experiments. Second, we assume that projections vary smoothly as we change the source voxel, that is, the columns of *W* are smooth functions of the column voxel. However, we do not explicitly assume that the incoming projections to a target voxel vary smoothly as we move the target voxel, or smoothness in the rows. Smoothness in target space leads to dependencies among the output variables of the multivariate regression problem, making it a so-called *structured* regression problem, which is generally more difficult to solve. Note that, because the data tend to produce patterns of projections that are spatially smooth, and because we enforce smoothness in the source space, some target smoothness will arise naturally.

#### Nadaraya-Watson connectome estimator.

With these simplifying assumptions, we can now state the model. Our data are now the pairs of center of mass voxels *c*_*e*_ and normalized injection densities ${\overset{-}{Y}}_{:,e}$, which we assume arise from an injection of one unit of virus to the center of mass. Kernel regression is a standard nonparametric method for estimating a smooth univariate or multivariate function. For simplicity, we use the Nadaraya-Watson estimator (Nadaraya, 1964; Watson, 1964) to estimate the connectivity:

$$W_{ij}=\frac{\underset{e:c_{e}\in S_{k}}{\sum }K(∥v_{j}-c_{e}∥){\overset{-}{Y}}_{ie}}{\underset{f:c_{f}\in S_{k}}{\sum }K(∥v_{j}-c_{f}∥)}=\underset{e:c_{e}\in S_{k}}{\sum }{\overset{-}{Y}}_{ie}\alpha_{ej}\;,$$ (1)

where

$$\alpha_{ej}=\frac{K(∥v_{j}-c_{e}∥)}{\underset{f:c_{f}\in S_{k}}{\sum }K(∥v_{j}-c_{f}∥)}\;,$$ (2)

and *k* is the unique index such that *v*_*i*_ ∈ 𝒮_*k*_, that is, we only average over injections in the major brain division containing the source voxel. Furthermore, we can construct the matrices

$$\begin{array}{ll} \overset{-}{Y} & =\left[{\overset{-}{Y}}_{:,1},{\overset{-}{Y}}_{:,2},\ldots ,{\overset{-}{Y}}_{:,m}\right]\\ A & =\left[\alpha_{ej}\right], \end{array}$$

so that the connectome is written compactly as a rank *m* matrix $W=\overset{-}{Y}A$, where $\overset{-}{Y}$ ∈ ℝ^*n*×*m*^ and *A* ∈ ℝ^*m*×*n*^. Note that each column in *A*, the coefficients *α*_*ej*_, has entries that sum to 1.

The Nadaraya-Watson estimator, Equation (1), has a number of nice properties: It does not require any fitting, because the coefficients *α*_*ej*_ are given explicitly in terms of the center of masses and kernel, Equation (2). For nonnegative data, it will produce a nonnegative connectivity matrix, which we require. Furthermore, it forms a compressed rank *m* representation of *W* that is only as large as the data. However, it does suffer some drawbacks; for example, it is well known that the Nadaraya-Watson estimator is biased for data that are not sampled uniformly and near boundaries.

Note also that experiments with center of masses *c*_*e*_ ∈ 𝒮_*k*_ do not have any influence outside of 𝒮_*k*_. This is because we do not want to average over experiments in vastly different brain areas. Therefore, the coefficients *α*_*e*,*k*_ are decoupled across major brain divisions. Essentially, we fit a different model for each major brain division.

#### Choice of spatial kernel.

We use a Gaussian radial basis function kernel:

$$K_{\sigma }(d)=\exp\left(-\frac{d^{2}}{2\sigma^{2}}\right),$$ (3)

where *σ* > 0 is a hyperparameter setting the length scale or bandwidth of the kernel function. The length scale *σ* is fit using nested cross-validation in the model selection phase. We search over *σ* ∈ [4, 50] in 11 logarithmically spaced increments, where *σ* is in units of 100 μm voxels. Note that while the kernel, Equation 3, has infinite support, we do not evaluate it on points outside the coarse structure of interest.

#### Evaluating performance via cross-validation.

To evaluate the performance of the model, we employ nested leave-one-out cross-validation. In the inner loop, we fit *m* − 1 different models on sets of *m* − 2 experiments in order to perform model selection, wherein we fit the hyperparameter *σ* of the kernel function *K*. The best model is then evaluated against the held-out experiment from the outer loop, and this process is repeated *m* times. The performance metric we choose to use is mean square error relative to the average squared norm of the prediction and left-out data:

$${\text{MSE}}_{\text{rel}}=\frac{2∥Y^{\text{pred}}-Y^{\text{true}}{∥}_{F}^{2}}{∥Y^{\text{pred}}{∥}_{F}^{2}+∥Y^{\text{true}}{∥}_{F}^{2}}.$$ (4)

This choice of normalization prevents experiments with small ∥*Y* ∥ from dominating the error, more heavily weighting experiments with larger signal (K. D. Harris et al., 2016).

The relative error in Equation (4) is approximately equal to the usual relative mean square error ∥*Y*^pred^ − *Y*^true^∥_*F*_^2^/∥*Y*^true^∥_*F*_^2^ when *Y*^pred^ is close to *Y*^true^, and this is not too small. To see this, let *Y*^pred^ = *Y*^true^ + *δ* where ∥*δ*∥_*F*_ ≤ *ϵ* and ∥*Y*^true^∥_*F*_ = *O*(1). Then, dropping the superscript “true” for clarity,

$${\text{MSE}}_{\text{rel}}=\frac{2∥\delta {∥}_{F}^{2}}{∥Y+\delta {∥}_{F}^{2}+∥Y{∥}_{F}^{2}}=\frac{2∥\delta {∥}_{F}^{2}}{2∥Y{∥}_{F}^{2}+∥\delta {∥}_{F}^{2}}=\frac{∥\delta {∥}_{F}^{2}}{∥Y{∥}_{F}^{2}}\left(\frac{1}{1+\frac{∥\delta {∥}_{F}^{2}}{2∥Y{∥}_{F}^{2}}}\right)=\frac{∥\delta {∥}_{F}^{2}}{∥Y{∥}_{F}^{2}}\left(1-O(\epsilon^{2})\right).$$

However, if *Y* is close to 0, our metric can be different. For example, if *Y*^pred^ = 1 and *Y*^true^ = 0.25, then MSE_rel_ = 2(1 − 0.25)^2^/(1^2^ + (0.25)^2^) = 106%. The usual relative mean square error would be (1 − 0.25)^2^/(0.25)^2^ = 900%. If either *Y*^true^ or *Y*^pred^ is 0 and the other is not, then MSE_rel_ = 200%, its maximum value.

Consider a set of experiments ${\left\{(c_{e},{\overset{-}{Y}}_{:,e})\right\}}_{e=1}^{m}$, where *c*_*e*_ is the center of mass of the *e*th injection. Let *C* ∈ ℝ^*n*×*m*^ be the matrix of injection center indicators, with entries *C*_*ie*_ = 1_{*c*_*e*_=*v*_*i*_}_. Define the kernel matrix *A*^*c*^ ∈ ℝ^*m*×*m*^ as the kernel evaluated at the centers of mass; then this is just *A*_*c*_ = *AC*. Thus the model prediction of the center of mass projections is $WC=\overset{-}{Y}AC=\overset{-}{Y}A_{c}$.

We can perform leave-one-out cross-validation efficiently after computing the coefficients *A*_*c*_ for a given set of data. If we leave out experiment *e*, the new model *W*^(−*e*)^ predicts that the projections from *c*_*e*_ are $Ŷ=W^{\left(-e\right)}C_{:,e}=\overset{-}{Y}A_{c}^{\left(-e\right)}$, where *A*_*c*_^(−*e*)^ has the *e*th diagonal entry equal to 0 and the corresponding column renormalized to sum to 1. Therefore,

$${\left(A_{c}^{\left(-e\right)}\right)}_{ij}=\left\{\begin{array}{cc} {\left(A_{c}\right)}_{ij}, & \;j\neq e,\\ \frac{{\left(A_{c}\right)}_{ij}}{1-{\left(A_{c}\right)}_{ee}}, & \;j=e\;\text{and}\;i\neq j,\\ 0, & i=j=e. \end{array}\right.$$

Extending the above result to compute the leave-one-out predictions for all of the experiments, we find that these are equal to $\overset{-}{Y}A_{c}^{\text{CV}}$, where

$${\left(A_{c}^{\text{CV}}\right)}_{ij}=\left\{\begin{array}{cc} \frac{{\left(A_{c}\right)}_{ij}}{1-{\left(A_{c}\right)}_{jj}}, & \;i\neq j,\\ 0, & \;i=j. \end{array}\right.$$

Thus, once we compute the coefficients *A*_*c*_, we set the diagonals equal to 0 and renormalize the columns to obtain *A*_*c*_^CV^. The leave-one-out cross-validation relative error of the voxel model is then

$$\frac{2∥Ŷ-\overset{-}{Y}{∥}_{F}^{2}}{∥Ŷ{∥}_{F}^{2}+∥\overset{-}{Y}{∥}_{F}^{2}},$$ (5)

where $Ŷ=\overset{-}{Y}A_{c}^{\text{CV}}$ are the leave-one-out predictions.

### Regionalized and Homogeneous Models

The application of Equation (1) results in a very large *n* × *n* voxel-scale connectivity matrix. Recall that we defined a parcellation of the brain into *r* regions 𝓡. We would like to be able to compare this with extant regional connectomes, which are smaller *r* × *r* matrices. With this, we can define the regional projection matrix, Π ∈ ℝ^*r*×*n*^, with entries:

$$\Pi_{ij}=1_{\left\{v_{j}\in R_{i}\right\}}.$$

That is, the *i*th row of Π has ones in entries corresponding to voxels in region *i*. Therefore, for some vector *x* ∈ ℝ^*n*^ corresponding to a voxel image of the brain, the vector *x*^R^ = Π *x* has entries corresponding to the sum of *x* over regions. Furthermore, consider another matrix Π^†^ ∈ ℝ^*n*×*r*^, with entries:

$$\Pi_{ij}^{†}=\frac{1_{\left\{v_{i}\in R_{j}\right\}}}{\left|R_{j}\right|},$$

where |𝓡_*j*_| is the number of voxels in region *j*. Then Π^†^, operating from the left on a length *r* vector, spreads the entries evenly over all of the voxels in a given region. Operating from the right, it averages over the voxels in a region. Note that ΠΠ^†^ = *I*_*r*_, so it is a right inverse of Π and in fact is a Moore-Penrose pseudoinverse.

With this notation, it becomes simple to convert voxel vectors and matrices into regional ones. We refer to the sum of the connection weights between two regions as the *connection strength* between the regions. Thus, the regional connection strength is given by

$$W^{\text{R}}=\Pi \;W\;\Pi^{⊺}.$$

However, these regions may be vastly different sizes, in which case a measure normalized by source and/or target region size is more appropriate. We define the *normalized projection density* as the connection strength between two regions divided by the size of the source and target region. In this case, the matrix becomes

$$W^{\text{R,norm density}}=\Pi^{†⊺}\;W\;\Pi^{†}.$$

Finally, our last normalization only normalizes by the size of the source region, which we call *normalized connection strength*:

$$W^{\text{R,norm strength}}=\Pi \;W\;\Pi^{†}.$$

This normalization is necessary to compare directly with the homogeneous model.

#### Fitting a homogeneous regional model.

As in Oh et al. (2014), one could also fit a regional model where connection strengths are fixed across regions by working directly with data that have been regionalized. We performed this for comparison and refer to the result as the *homogeneous* model. Let *X*^R^ = Π *X* and *Y*^R^ = Π *Y*. Then the model fit to these regional data is found via nonnegative least squares as

$$W^{\text{homog}}=\arg\underset{W^{\prime }\geq 0}{\min}∥W^{\prime }X^{\text{R}}-Y^{\text{R}}{∥}_{F}^{2}.$$ (6)

Note that the output *W*^homog^ is a normalized connection strength, since entry (*i*, *j*) is the expected volume of fluorescence in region *i* per unit of virus in region *j*.

#### Comparing the regionalized voxel model to the homogeneous model.

Let $Ŷ\in R^{n}$ be the voxel prediction; we can compute the regionalized prediction ${Ŷ}^{\text{R}}\in R^{r}$ by projecting the voxel predictions into the regional space: ${Ŷ}^{\text{R}}=\Pi Ŷ$. It is important to note that although the comparison between the regionalized voxel model and the homogeneous model are done in the same space ℝ^*r*^, the predictions themselves are slightly different. The regionalized voxel predictions ${Ŷ}_{:,e}^{\text{R}}$ are the predicted result of a unit injection into the center of mass of the injection *c*_*e*_, whereas the regional prediction ${Ŷ}_{:,e}^{R}=W^{\text{homog}}X_{:,e}$ is the regional prediction of the projection from the full injection *X*_:,*e*_.

#### Manual checks of regional projections.

To check individual experiments for segmentation errors, we used the interactive projection experiment detail view page of the Allen Mouse Brain Connectivity Atlas (e.g., http://connectivity.brain-map.org/projection/experiment/100141219). We first set the “projection volume” threshold to 0 so that all targets were displayed, then selected the bar graph for all ipsilateral and contralateral targets to align the raw image viewer for each. The viewer automatically centers on the area with the highest signal in each anatomical region. However, when no projections were initially apparent we checked several sections rostral and caudal to the initial location as well as the surrounding region. Target regions that contained eGFP-expressing axons were labeled 1 (true positive). If no projections were observed the target was assigned a 0 (true negative). In cases where there were a small number of axons that appeared to be passing fibers without branches or boutons, we assigned the target a 0. Most segmentation errors were caused by tissue edges or blood vessels that were detected by the automatic segmentation algorithm. See Figure S1 (Knox et al., 2019). The following manually validated experiments were used to compare model weights to experimental data: 100141219, 100147853, 309113907, 479756361, 500836840, 500837552, 522409371, 546103149, 112424813, 117298988, 126908007, and 180916954. The first eight are the VISp injections plotted in Figure 7.

## ACKNOWLEDGMENTS

This work was supported by the Allen Institute for Brain Science. The authors wish to thank the Allen Institute founder, Paul G. Allen, for his vision, encouragement, and support. Additionally, the project described was supported in part by the National Institute on Aging of the National Institutes of Health. Its contents are solely the responsibility of the authors and do not necessarily represent the official views of the National Institutes of Health. We would like to thank Lydia Ng and Nathan Gouwens for assistance with the cortical flattening.

## SUPPORTING INFORMATION

All of the data used to construct these models are available at http://connectivity.brain-map.org. The data used in this analysis were cached in March 2017. The curved coordinate system is described in http://help.brain-map.org/download/attachments/2818171/Mouse_Common_Coordinate_Framework.pdf, with the specific data available from http://download.alleninstitute.org/informatics-archive/current-release/mouse_ccf/cortical_coordinates/ccf_2017. All code used to build this model is available from https://github.com/AllenInstitute/mouse_connectivity_models (Knox, 2018).

## AUTHOR CONTRIBUTIONS

Joseph E. Knox: Formal analysis; Investigation; Methodology; Software; Visualization; Writing – original draft; Writing – review & editing. Kameron Decker Harris: Conceptualization; Formal analysis; Investigation; Methodology; Software; Visualization; Writing – original draft; Writing – review & editing. Nile Graddis: Software; Writing – review & editing. Jennifer D. Whitesell: Validation; Visualization; Writing – original draft; Writing – review & editing. Hongkui Zeng: Supervision. Julie A. Harris: Data curation; Supervision; Validation; Writing – review & editing. Eric Shea-Brown: Conceptualization; Supervision; Writing – review & editing. Stefan Mihalas: Conceptualization; Supervision; Writing – original draft; Writing – review & editing.

## FUNDING INFORMATION

Julie A. Harris, National Institute on Aging (http://dx.doi.org/10.13039/100000049), Award ID: R01AG047589. Kameron Decker Harris, Directorate for Mathematical and Physical Sciences (http://dx.doi.org/10.13039/100000086), Award ID: 1122106. Eric Shea-Brown, Directorate for Mathematical and Physical Sciences (http://dx.doi.org/10.13039/100000086), Award ID: 1514743. Kameron Decker Harris, Big Data for Genomics & Neuroscience Training Grant, Award ID: 1T32CA206089-01A1.

## TECHNICAL TERMS

Voxel: A 3-D cubic volume element; the generalization of a pixel.
Major brain divisions: The set of 12 major brain divisions from the 3-D Allen Mouse Brain Reference Atlas: isocortex, olfactory, areas, hippocampus, cortical subplate, striatum, pallidum, thalamus, hypothalamus, midbrain, pons, medulla, and cerebellum (also called *coarse structures*).
Connection strength: The sum of the connection weights from all voxels in a source region 𝓡_1_ to all voxels in a target region 𝓡_2_, denoted *W*_21_^R^.
Coarse structures: See *major brain divisions*.
Regions: The set of 291 intermediate brain structures from 3-D Allen Mouse Brain Reference Atlas (also called *summary structures*).
Normalized connection density: The connection strength from 𝓡_1_ to 𝓡_2_ divided by the product of their sizes: $\frac{W_{21}^{\text{R}}}{\left|R_{1}||R_{2}\right|}$.
Wild type mouse (C57BL/6J): Mice of strain C57BL/6J that have not been genetically altered.
Radial basis function: A monotonically decreasing, nonnegative function of distance.
Frobenius norm: The two norm of a matrix viewed as a vector: $∥A{∥}_{F}=\sqrt{\sum_{ij}A_{ij}^{2}}$.
Normalized connection strength: The connection strength from 𝓡_1_ to 𝓡_2_ divided by the size of the source region: $\frac{W_{21}^{\text{R}}}{\left|R_{1}\right|}$.

## References

[bib1] Bock D. D., Lee W.-C. A., Kerlin A. M., Andermann M. L., Hood G., Wetzel A. W., … Reid R. C. (2011). Network anatomy and in vivo physiology of visual cortical neurons. Nature, 471(7337), 177–182. 21390124 10.1038/nature09802 PMC3095821

[bib2] Bohland J. W., Wu C., Barbas H., Bokil H., Bota M., Breiter H. C., … Mitra P. P. (2009). A proposal for a coordinated effort for the determination of brainwide neuroanatomical connectivity in model organisms at a mesoscopic scale. PLoS Computational Biology, 5(3), e1000334 19325892 10.1371/journal.pcbi.1000334 PMC2655718

[bib3] Bota M., Dong H.-W., & Swanson L. W. (2003). From gene networks to brain networks. Nature Neuroscience, 6(8), 795–799. 12886225 10.1038/nn1096

[bib4] Ercsey-Ravasz M., Markov N. T., Lamy C., Van Essen D. C., Knoblauch K., Toroczkai Z., & Kennedy H. (2013). A predictive network model of cerebral cortical connectivity based on a distance rule. Neuron, 80(1), 184–197. 24094111 10.1016/j.neuron.2013.07.036 PMC3954498

[bib5] Felleman D. J., & Van Essen D. C. (1991). Distributed hierarchical processing in the primate. Cerebral Cortex, 1(1), 1–47. 1822724 10.1093/cercor/1.1.1-a

[bib6] Gămănuţ R., Kennedy H., Toroczkai Z., Ercsey-Ravasz M., Van Essen D. C., Knoblauch K., & Burkhalter A. (2018). The mouse cortical connectome, characterized by an ultra-dense cortical graph, maintains specificity by distinct connectivity profiles. Neuron, 97(3), 698–715.e10. 29420935 10.1016/j.neuron.2017.12.037 PMC5958229

[bib7] Glickfeld L. L., Andermann M. L., Bonin V., & Reid R. C. (2013). Cortico-cortical projections in mouse visual cortex are functionally target specific. Nature Neuroscience, 16(2), 219–226. 23292681 10.1038/nn.3300 PMC3808876

[bib8] Harris J. A., Mihalas S., Hirokawa K. E., Whitesell J. D., Knox J., Bernard A., … Zeng H. (2018). The organization of intracortical connections by layer and cell class in the mouse brain. bioRxiv:292961.

[bib9] Harris K. D., Mihalas S., & Shea-Brown E. (2016). High resolution neural connectivity from incomplete tracing data using nonnegative spline regression. In Lee D. D., Sugiyama M., Luxburg U. V., Guyon I., & Garnett R. (Eds.), Advances in Neural Information Processing Systems 29.

[bib10] Jenett A., Rubin G. M., Ngo T.-T. B., Shepherd D., Murphy C., Dionne H., … Zugates C. T. (2012). A GAL4-driver line resource for *Drosophila* neurobiology. Cell Reports, 2(4), 991–1001. 23063364 10.1016/j.celrep.2012.09.011 PMC3515021

[bib11] Kleinfeld D., Bharioke A., Blinder P., Bock D. D., Briggman K. L., Chklovskii D. B., … Sakmann B. (2011). Large-scale automated histology in the pursuit of connectomes. Journal of Neuroscience, 31(45), 16125–16138. 22072665 10.1523/JNEUROSCI.4077-11.2011 PMC3758571

[bib12] Knox J. (2018). Python package providing mesoscale connectivity models for mouse, Github, https://github.com/AllenInstitute/mouse_connectivity_models

[bib13] Knox J. E., Decker Harris K., Graddis N., Whitesell J. D., Zeng H., Harris J. A., … Mihalas S. (2019). Supporting information for “High-resolution data-driven model of the mouse connectome.” Network Neuroscience, 3(1), 217–236. 10.1162/netn_a_00066 PMC6372022 30793081

[bib14] Kuan L., Li Y., Lau C., Feng D., Bernard A., Sunkin S. M., … Ng L. (2015). Neuroinformatics of the Allen Mouse Brain Connectivity Atlas. Methods, 73, 4–17. 25536338 10.1016/j.ymeth.2014.12.013

[bib15] Laramée M.-E., & Boire D. (2015). Visual cortical areas of the mouse: Comparison of parcellation and network structure with primates. Frontiers in Neural Circuits, 8, 149 25620914 10.3389/fncir.2014.00149 PMC4286719

[bib16] Majka P., Chaplin T. A., Yu H.-H., Tolpygo A., Mitra P. P., Wójcik D. K., & Rosa M. G. P. (2016). Towards a comprehensive atlas of cortical connections in a primate brain: Mapping tracer injection studies of the common marmoset into a reference digital template. Journal of Comparative Neurology, 524(11), 2161–2181. 27099164 10.1002/cne.24023 PMC4892968

[bib17] Markov N. T., Ercsey-Ravasz M. M., Ribeiro Gomes A. R., Lamy C., Magrou L., Vezoli J., … Kennedy H. (2014). A weighted and directed interareal connectivity matrix for macaque cerebral cortex. Cerebral Cortex, 24(1), 17–36. 23010748 10.1093/cercor/bhs270 PMC3862262

[bib18] Nadaraya E. A. (1964). On estimating regression. Theory of Probability and Its Applications, 9(1), 141–142.

[bib19] Oh S. W., Harris J. A., Ng L., Winslow B., Cain N., Mihalas S., … Zeng H. (2014). A mesoscale connectome of the mouse brain. Nature, 508(7495), 207–214. 24695228 10.1038/nature13186 PMC5102064

[bib20] Ragan T., Kadiri L. R., Venkataraju K. U., Bahlmann K., Sutin J., Taranda J., … Osten P. (2012). Serial two-photon tomography for automated ex vivo mouse brain imaging. Nature Methods, 9(3), 255–258. 22245809 10.1038/nmeth.1854 PMC3297424

[bib21] Rubinov M., Ypma R. J. F., Watson C., & Bullmore E. T. (2015). Wiring cost and topological participation of the mouse brain connectome. Proceedings of the National Academy of Sciences, 112(32), 10032–10037. 10.1073/pnas.1420315112 PMC4538676 26216962

[bib22] Sethi S. S., Zerbi V., Wenderoth N., Fornito A., & Fulcher B. D. (2017). Structural connectome topology relates to regional BOLD signal dynamics in the mouse brain. Chaos: An Interdisciplinary Journal of Nonlinear Science, 27(4), 047405 10.1063/1.4979281 28456172

[bib23] Shih C.-T., Sporns O., Yuan S.-L., Su T.-S., Lin Y.-J., Chuang C.–C., … Chiang A.–S. (2015). Connectomics-based analysis of information flow in the *Drosophila* brain. Current Biology, 25(10), 1249–1258. 25866397 10.1016/j.cub.2015.03.021

[bib24] Sporns O. (2010). Networks of the brain. Cambridge, MA: MIT Press.

[bib25] Stafford J. M., Jarrett B. R., Miranda-Dominguez O., Mills B. D., Cain N., Mihalas S., … Fair D. A. (2014). Large-scale topology and the default mode network in the mouse connectome. Proceedings of the National Academy of Sciences, 111(52), 18745–18750. 10.1073/pnas.1404346111 PMC4284535 25512496

[bib26] Wahba G. (1990). Spline models for observational data. Philadelphia, PA: SIAM.

[bib27] Wang Q., Sporns O., & Burkhalter A. (2012). Network analysis of corticocortical connections reveals ventral and dorsal processing streams in mouse visual cortex. Journal of Neuroscience, 32(13), 4386–4399. 22457489 10.1523/JNEUROSCI.6063-11.2012 PMC3328193

[bib28] Wang X.-J., & Kennedy H. (2016). Brain structure and dynamics across scales: In search of rules. Current Opinion in Neurobiology, 37(Suppl. C), 92–98. 26868043 10.1016/j.conb.2015.12.010 PMC5029120

[bib29] Watson G. S. (1964). Smooth regression analysis. Sankhyā: The Indian Journal of Statistics, Series A (1961–2002), 26(4), 359–372.

[bib30] White J. G., Southgate E., Thomson J. N., & Brenner S. (1986). The structure of the nervous system of the nematode *Caenorhabditis elegans*. Philosophical Transactions of the Royal Society of London B: Biological Sciences, 314(1165), 1–340. 22462104 10.1098/rstb.1986.0056

[bib31] Ypma R. J. F., & Bullmore E. T. (2016). Statistical analysis of tract-tracing experiments demonstrates a dense, complex cortical network in the mouse. PLoS Computational Biology, 12(9), e1005104 27617835 10.1371/journal.pcbi.1005104 PMC5019374

[bib32] Zingg B., Hintiryan H., Gou L., Song M., Bay M., Bienkowski M., … Dong H.-W. (2014). Neural networks of the mouse neocortex. Cell, 156(5), 1096–1111. 24581503 10.1016/j.cell.2014.02.023 PMC4169118

