# Supplementary material for: High-resolution data-driven model of the mouse connectome
Source: Netw Neurosci. 2018 Dec 1;3(1):217–36. doi: 10.1162/netn_a_00066 (PMC6372022; doi:10.1162/netn_a_00066)
Supplement: Supplementary file 1 [file netn-03-217-s001.pdf]

Knox, J. E., Harris, K. D., Graddis, N., Whitesell, J. D., Zeng, H., Harris, J. A., Shea-Brown, E., & Mihalas, S. (2019). High resolution datadriven model of the mouse connectome. *Network Neuroscience. 3*(1), 217–236. https://doi.org/10.1162/netn_a_00066

Supplement to "High resolution data-driven model of the mouse connectome"

Joseph E. Knox, Kameron Decker Harris, Nile Graddis, Jennifer D. Whitesell, Hongkui Zeng, Julie A. Harris, Eric Shea-Brown, and Stefan Mihalas.
Network Neuroscience. 2018. doi: 10.1162/netn_a_00066

Model extensions

In addition to the model previously described in the Methods, we describe a few extensions which were also tested.

Fitting with the entire injection volume

We described an efficient algorithm for computing the leave-one-out model estimate in the Methods section. Alternatively, one could compute the leave-one-out estimate using the full injection volume: $\hat{Y} = W^{(-e)}X$. However, not only is this procedure orders of magnitudes slower, but this model variant performs only marginally better (by about 1%) in a small number of major brain divisions and worse (by 1–10%) in others. See Table S6.

Homogeneous model with higher resolution data

In Table 1, we compared our voxel-scale model to a homogeneous model where both used 100 µm data. We have also fit the homogeneous regional model directly to 10 µm data. In Table S7, we observe that the homogeneous model performs better than the 100 µm voxel regionalized model when it is constructed this way. However, it is possible that the voxel model will also improve if the 10 µm data are used directly to construct it. We leave such an extension for future work.

Log-model and variance stabilization

For data containing multiplicative or Poisson noise, the variance can scale with the mean. Our projection densities are derived from counts of fluorescent pixels, thus it is reasonable to expect some kind of multiplicative noise in these data. In order to stabilize the variance, we have applied the log-transform to the normalized projection density data $\bar{Y}_{ie}$ before fitting. This is done via the transformation $f(\cdot) = \log(\cdot + \epsilon)$, where $\epsilon = 10^{-8}$ is set to approximately the fifth percentile of the normalized projection densities. The relative test and training errors are presented in Table S8, as well as the relative errors of the log-transformed standard model (Methods). The difference in performance of the two models shows that, if the log-transformed weights are desired, then fitting the model directly to log-transformed data may be more appropriate. Furthermore, the relative errors in log-space are much smaller. The log-transform weights weak projections much more than in the raw data, and strong projections less. However, the interpretation of the weights is quite different since the units are changed.

Target post-smoothing

Our voxel model smooths in the source domain, i.e. the columns of W or rows of $W^\intercal$ (Fig. 2). The method interpolates between injection centers of mass (Eqn. 1), resulting in projection patterns from neighboring source voxels that are highly correlated. It should be noted however that each of these projection patterns are not necessarily smooth as there is no smoothing in the target domain, as opposed to the method in K. D. Harris et al. (2016) where this is enforced. In other words, the output from neighboring voxels in the connectivity is highly correlated, but the input into neighboring voxels is not, and that could possibly be an important assumption. Our method has no way of imposing smoothing in the target domain, but instead we have tried post-hoc smoothing in the target space. We have chosen a median smoother with a window size of 500 µm, close to the average injection center distance (Table S2). Additionally, we constrained the domain of the target smoothing to the level of individual regions in order to preserve the regionalized connectivity.

Visual comparison of model extensions

A visual comparison of the standard model and the variants using log-transformation or post-smoothing is shown in Figure S2. For the log model, the weights are then transformed back into the original space via $f^{-1}(\cdot)$ after fitting.

Abbreviation	Full structure name
FRP	Frontal pole, cerebral cortex
MOs	Secondary motor area
ACAd	Anterior cingulate area, dorsal part
ACAv	Anterior cingulate area, ventral part
PL	Prelimbic area
ILA	Infralimbic area
ORBl	Orbital area, lateral part
ORBm	Orbital area, medial part
ORBvl	Orbital area, ventrolateral part
AId	Agranular insular area, dorsal part
AIv	Agranular insular area, ventral part
AIp	Agranular insular area, posterior part
GU	Gustatory areas
VISC	Visceral area
SSs	Supplemental somatosensory area
SSp-bfd	Primary somatosensory area, barrel field
SSp-tr	Primary somatosensory area, trunk
SSp-ll	Primary somatosensory area, lower limb
SSp-ul	Primary somatosensory area, upper limb
SSp-un	Primary somatosensory area, unassigned
SSp-n	Primary somatosensory area, nose
SSp-m	Primary somatosensory area, mouth
MOp	Primary motor area
VISal	Anterolateral visual area
VISl	Lateral visual area
VISp	Primary visual area
VISpl	Posterolateral visual area
VISli	Laterointermediate area
VISpor	Postrhinal area
VISrl	Rostrolateral visual area
VISa	Anterior area
VISam	Anteromedial visual area
VISpm	posteromedial visual area
RSPagl	Retrosplenial area, lateral agranular part
RSPd	Retrosplenial area, dorsal part
RSPv	Retrosplenial area, ventral part
AUDd	Dorsal auditory area
AUDp	Primary auditory area
AUDpo	Posterior auditory area
AUDv	Ventral auditory area
TEa	Temporal association areas
ECT	Ectorhinal area
ENTl	Entorhinal area, lateral part

Table S1: Structure abbreviations and names for Isocortical regions with the inclusion of the Entorhinal area, lateral part of the Hippocampus.

The pictures are top-view projections in which the projection signal has been summed through the superior-inferior axis. We show the predicted connectivity weights for the voxel located at the center of mass of an injection volume for a number of experiments, as well as the raw data (normalized projection densities) for those experiments. Note that these experiments were used in the fitting procedure, so this depicts training error or goodness of fit, not test error or generalization. In all cases, the projection patterns look qualitatively the same, although in some cases (ENTI, AON) the log-transformed model seems to put less weight onto nearby injections.

**Major division**	**Expts.**	**CCF Regions**	**Multiple expts.**	**Total vol. (mm^3)**	**Mean reg. vol. (mm^3)**	**Mean inj. vol. (mm^3)**	**Inj. dist. (mm)**
**Isocortex**	126	43	37	61.88	1.44	0.23	0.51
**Olfactory Areas**	21	11	7	23.28	1.89	0.12	0.71
**Hippocampus**	43	12	9	21.35	1.68	0.12	0.51
**Cortical Subplate**	8	7	2	4.43	0.61	0.15	0.88
**Striatum**	26	14	7	22.61	1.52	0.16	0.67
**Palidum**	10	9	2	4.75	0.47	0.12	0.61
**Thalamus**	43	40	25	10.30	0.24	0.11	0.40
**Hypothalamus**	34	41	14	7.64	0.15	0.33	0.41
**Midbrain**	43	33	12	18.66	0.46	0.21	0.46
**Pons**	16	21	7	8.51	0.30	0.15	0.60
**Medulla**	39	43	18	15.74	0.30	0.23	0.49
**Cerebellum**	19	17	7	27.22	1.57	0.08	0.76

Table S2: Summary statistics for included data. "Multiple expts." is the number of regions containing multiple experiments. "Inj. dist." is the mean distance from any voxel to an injection center of mass.

Division	Hemisphere	BIC Lognormal	Inv. Gamma	Exponential	Normal
Whole-Brain	Ipsilateral	-1.61e6	-1.3986	-1.09e6	6.35e23
	Contralateral	-1.78e6	-1.57e6	-1.28e6	6.33e24
Isocortex	Ipsilateral	-2.71e4	-2.59e4	-2.57e4	-2.16e4
	Contralateral	-3.38e4	-3.27e4	-3.18e4	-2.73e4

Table S3: Model selection for the best fit distribution for the connectivity weights. The best-fit model was chosen through minimizing the BIC. The weight distributions of both ipsilateral and contralateral whole-brain and cortico-cortical connection weights were best fit by a lognormal distribution. However, the empirical weight distributions did not pass the Kolmogorov-Smirnov test at $\alpha = 0.05$.

Division	Projection Hemisphere	Component	μ	σ	weight
Whole-Brain	Ipsilateral	1	-6.27	0.27	0.297
		2	-5.04	0.22	0.330
		3	-7.72	0.82	0.078
		4	-25.61	56.87	0.020
		5	-12.16	7.69	0.011
		6	-3.81	0.26	0.297
	Contralateral	1	-4.37	0.28	0.269
		2	-6.80	0.27	0.281
		3	-5.59	0.21	0.360
		4	-12.77	5.61	0.012
		5	-35.04	31.69	0.001
		6	-8.35	0.83	0.074
		7	-22.36	15.62	0.003
Isocortex	Ipsilateral	1	-5.25	0.27	0.217
		2	-4.22	0.14	0.435
		3	-3.23	0.16	0.348
	Contralateral	1	-5.57	0.37	0.500
		2	-4.22	0.31	0.500

Table S4: Fitted Gaussian mixture model parameters for the whole-brain and cortico-cortical logarithmically scaled connectivity weight distributions broken down by projection hemisphere. In both cases, the logarithmically transformed weights distributions failed to pass the Shapiro-Wilk test for normality, so we fit a mixture of Gaussians to each of the logarithmically transformed distributions. As in Table S3, the number of Gaussian components was selected to minimize the BIC.

		RMSE	
Division	Projection Hemisphere	Power Law	Exponential
Whole-Brain	Ipsilateral	3.76	3.79
	Contralateral	4.24	4.28
Isocortex	Ipsilateral	1.48	1.56
	Contralateral	1.96	1.99

Table S5: Model comparison of the best fit relation between normalized connection density and pairwise distances between anatomical regions. The power law and exponential relations were fit through a nonlinear optimization algorithm (Levenberg-Marquadt) to minimize the mean squared residuals. The performance of the two models is similar, with the power law relation having slightly lower root mean squared error (RMSE).

Major division	Voxel MSE_{rel}		Region MSE_{rel}		Region PTP	
**Isocortex**	65%	(16%)	33%	(9%)	31%	(8%)
**Olfactory Areas**	93%	(28%)	51%	(14%)	50%	(15%)
**Hippocampus**	92%	(21%)	56%	(17%)	53%	(19%)
**Cortical Subplate**	115%	(27%)	93%	(26%)	79%	(26%)
**Striatum**	103%	(7%)	47%	(3%)	41%	(2%)
**Pallidum**	118%	(33%)	81%	(30%)	54%	(20%)
**Thalamus**	113%	(10%)	76%	(8%)	71%	(10%)
**Hypothalamus**	64%	(18%)	48%	(16%)	41%	(15%)
**Midbrain**	88%	(10%)	44%	(5%)	39%	(5%)
**Pons**	102%	(30%)	68%	(24%)	63%	(23%)
**Medulla**	96%	(27%)	53%	(15%)	50%	(21%)
**Cerebellum**	174%	(3%)	83%	(2%)	68%	(1%)

Table S6: Errors of the standard model when using the total injection volume for fitting and evaluation.

Major division	Region MSE_{rel}		Region PTP	
**Isocortex**	31%	(17%)	28%	(15%)
**Olfactory Areas**	32%	(4%)	30%	(4%)
**Hippocampus**	38%	(41%)	35%	(41%)
**Cortical Subplate**	62%	(2%)	43%	(2%)
**Striatum**	39%	(12%)	40%	(13%)
**Pallidum**	57%	(2%)	42%	(2%)
**Thalamus**	33%	(6%)	30%	(5%)
**Hypothalamus**	40%	(3%)	49%	(3%)
**Midbrain**	40%	(12%)	33%	(13%)
**Pons**	58%	(11%)	56%	(12%)
**Medulla**	48%	(10%)	52%	(11%)
**Cerebellum**	60%	(2%)	29%	(3%)

Table S7: Cross-validated errors of a homogeneous model built with 10 μm data.

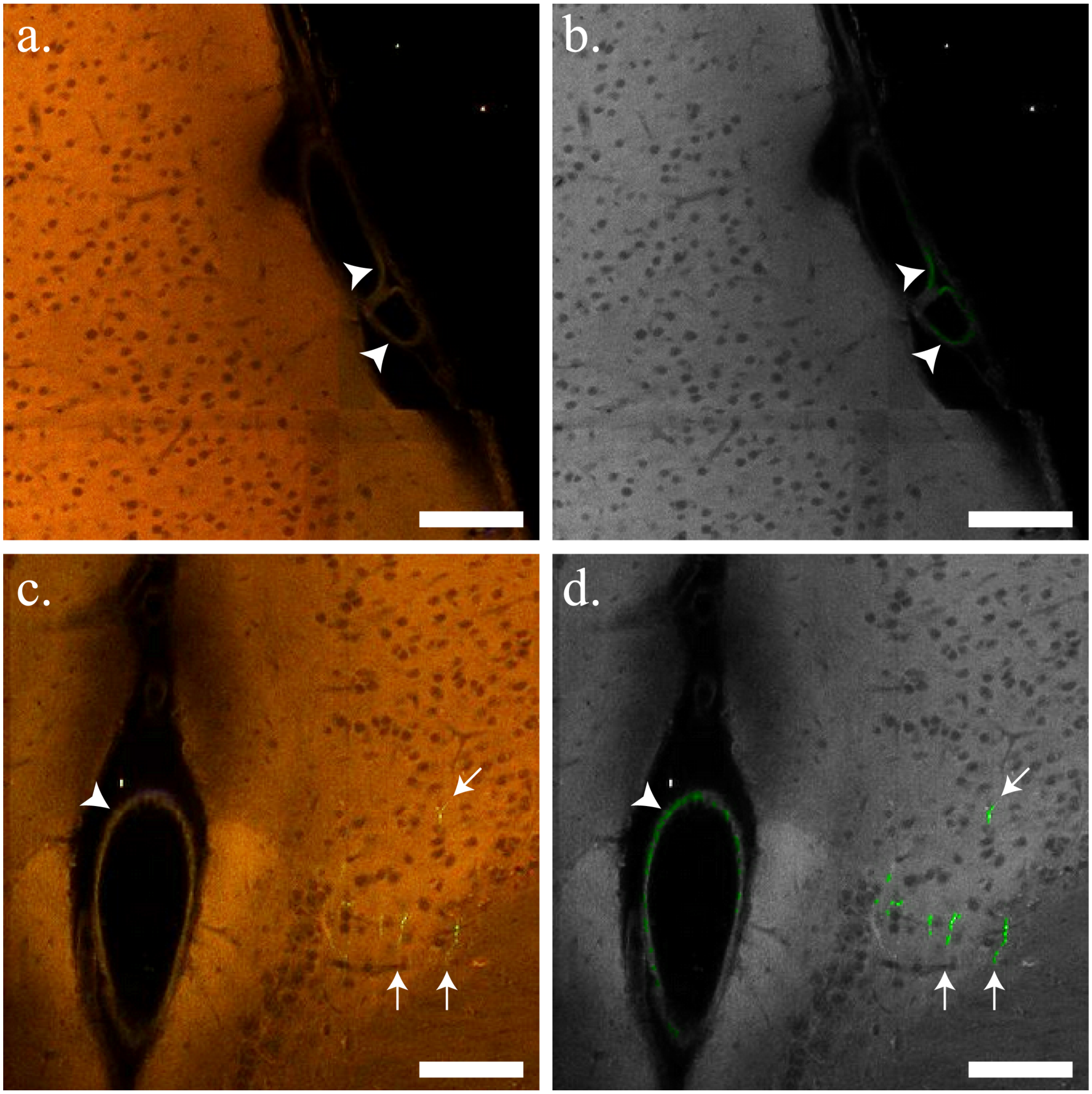

Figure S1: Segmentation artifacts in experiment 100147853, an injection delivered to source region VISp. Serial two-photon fluorescence images (a, c) and the same images in grayscale with automated segmentation overlaid in green (b, d). Arrowheads point to segmentation errors where blood vessels or tissue edges were detected by the segmentation algorithm. (a–b) The target region (SSs) was labeled a true negative. (c–d) A different target region (RSPv) that contained both false positive signal (arrowhead) and true positive signal corresponding to axonal projections (arrows). This target was labeled true positive. Scale bar = 100 µm.

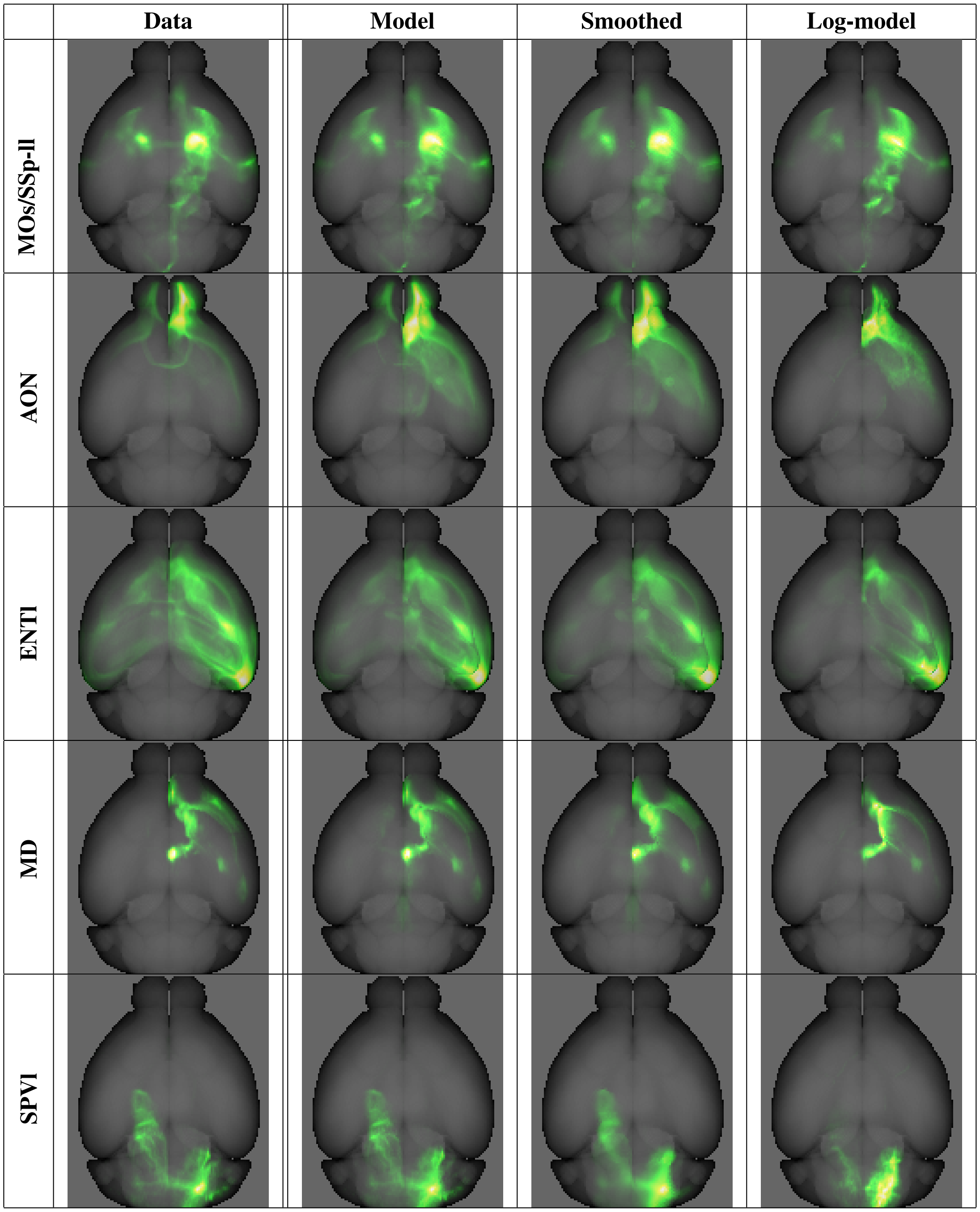

Figure S2: A comparison of the model extensions. Each image depicts the projections from the center of mass of an injection in a number of regions (MOs/SSp-II, AON 'anterior olfactory nucleus', ENTl, MD 'mediodorsal nucleaus of thalamus', SPVI 'spinal nucleus of the trigeminal, interpolar part' Medulla). The raw data are shown as well as the voxel model presented in the main text, the post-smoothed model, and the model built with log-transformed data. Differences between the various models and data reflect the training error (goodness of fit).

Table S8: Log-transformed model. We fit the model in the same manner as before but using log-transformed data $\log(\bar{Y}_{ie} + \epsilon)$, where $\epsilon = 10^{-8}$. Using the same relative error function, Eqn. 4, the nested leave-one-out errors for this log-transformed model are presented here. We also evaluate the relative error for log-transformed predictions of the standard model for comparison.

Major division	Model	Voxel MSE_{rel}		Region MSE_{rel}		Region PTP	
**Isocortex**	Log	2.0%	(1.2%)	0.8%	(0.4%)	0.8%	(0.4%)
	Standard	15.8%	(15.9%)	13.1%	(13.5%)	12.8%	(13.2%)
**Olfactory Areas**	Log	1.6%	(0.9%)	0.6%	(0.3%)	0.7%	(0.4%)
	Standard	5.7%	(5.5%)	4.3%	(4.3%)	4.3%	(4.3%)
**Hippocampus**	Log	1.7%	(0.5%)	0.5%	(0.2%)	0.6%	(0.2%)
	Standard	11.8%	(11.9%)	10.9%	(11.2%)	10.9%	(11.2%)
**Cortical Subplate**	Log	3.5%	(2.2%)	3.3%	(1.9%)	2.8%	(1.3%)
	Standard	7.7%	(6.7%)	7.6%	(7.2%)	7.0%	(6.5%)
**Striatum**	Log	1.4%	(0.8%)	0.4%	(0.2%)	0.4%	(0.3%)
	Standard	7.3%	(7.1%)	5.6%	(5.5%)	5.5%	(5.4%)
**Pallidum**	Log	2.4%	(0.6%)	1.0%	(0.2%)	0.6%	(0.2%)
	Standard	8.2%	(7.4%)	6.0%	(5.5%)	2.8%	(2.3%)
**Thalamus**	Log	2.0%	(1.0%)	0.8%	(0.4%)	0.8%	(0.4%)
	Standard	12.5%	(12.4%)	9.9%	(9.9%)	9.6%	(9.6%)
**Hypothalamus**	Log	1.9%	(1.3%)	0.5%	(0.3%)	0.4%	(0.3%)
	Standard	6.5%	(6.4%)	3.8%	(3.8%)	3.5%	(3.5%)
**Midbrain**	Log	1.7%	(1.0%)	0.4%	(0.2%)	0.4%	(0.2%)
	Standard	7.5%	(7.4%)	4.8%	(4.8%)	4.6%	(4.6%)
**Pons**	Log	2.6%	(1.4%)	0.6%	(0.3%)	0.4%	(0.2%)
	Standard	9.2%	(8.6%)	3.9%	(3.6%)	2.3%	(2.2%)
**Medulla**	Log	1.6%	(1.0%)	0.2%	(0.1%)	0.2%	(0.1%)
	Standard	5.8%	(5.7%)	1.8%	(1.8%)	1.6%	(1.6%)
**Cerebellum**	Log	1.0%	(0.8%)	0.2%	(0.1%)	0.1%	(0.1%)
	Standard	5.0%	(4.7%)	1.8%	(1.7%)	1.4%	(1.4%)
